# Non-fluent Primary Progressive Aphasia and Its Association to Motor Neuron Disease

**DOI:** 10.7759/cureus.66447

**Published:** 2024-08-08

**Authors:** Sarah Geevarughese, Rajul Parikh

**Affiliations:** 1 Clinical, Biomedical, and Educational Research, Edward Via College of Osteopathic Medicine, Spartanburg, USA; 2 Neurology, HCA Florida Memorial Hospital, Jacksonville, USA

**Keywords:** non-fluent variant primary progressive aphasia, frontotemporal lobar degeneration, motor neurone disease, frontotemporal dementia, primary progressive aphasia

## Abstract

Frontotemporal dementia (FTD) is among the most common forms of dementia, with an average symptom onset in the fifth decade of life. Neuropathologic changes in FTD demonstrate degeneration in the frontal and/or temporal lobes, which is defined as frontotemporal lobar degeneration (FTLD). FTD is categorized into a subset of variants by symptomatic presentation and corresponding clinical workup. Primary progressive aphasia (PPA) is among these variants of FTD and is distinguished by its primary clinical presentation of language impairment with correlating neuropathology in the aforementioned areas of the brain. More specifically, the classification of PPA is further subdivided into three clinical variants, which has allowed for appropriate diagnostic and prognostic considerations within this patient population. Among these variants in PPA are the semantic (svPPA), non-fluent (navPPA), and logopenic (lvPPA) forms. Motor neuron disease (MND) is a progressive and irreversible process of neuronal degeneration that can lead to an upper motor neuron, a lower motor neuron, or a combination of these two symptomologies. FTD and its association with MND is a well-established spectrum, although more rarely among the PPA variant of FTD. Comparatively, there is a significant body of clinical knowledge on the association between the behavioral variant of FTD (bvFTD) and MND. This is the case of a 69-year-old female with navPPA who later presented with clinical symptoms of MND. Although the two clinical diagnoses, PPA and MND, are irreversible and progressive, this case serves to elucidate diagnostic and prognostic considerations in this rare patient population.

## Introduction

Frontotemporal dementia (FTD) is a form of dementia that presents with a subset of clinical symptomology in an insidious and progressive manner. FTD is subdivided into two major variants: behavioral (bvFTD) and primary progressive aphasia (PPA). The most common variant is bvFTD, which is the variant diagnosed in approximately 50% of the FTD patient population. BvFTD is characterized by the symptom presentation of behavioral changes as an early clinical sign, which include but are not limited to disinhibition, compulsion, and apathy. Conversely, PPA is distinguished by the primary presentation of language deficits, which impair daily life [1].

In 2011, the classification system for PPA patients was modified to categorize the patient population into subgroups that better represented their clinical presentation. The value of identifying appropriate variants is that this leads to a clearer outlook on patient progression and treatment options. Three variants were identified in this modified classification: non-fluent (navPPA), semantic (svPPA), and logopenic (lvPPA). NavPPA is distinguished by its character of agrammatism, or difficulty with speech production, that presents as sudden interruptions in speech. SvPPA is characterized by its defining symptom of impaired semantic functioning. This includes difficulties in naming objects, loss of word comprehension, and overall progressive loss in the meaning of words [2]. LvPPA is distinguished from the other two variants in that it presents with pauses in word finding, impaired repetition, and deficits in comprehension. Declines in episodic memory are often associated with lvPPA, and therefore, this variant is often denoted as a subset of Alzheimer’s rather than PPA.

To make a diagnosis of PPA, patient history is vital, while neuroimaging and neuropsychological evaluation offer supportive evidence that excludes alternative etiologies. A diagnosis of PPA requires that the initial and most prominent clinical symptom interfering with daily functioning is language impairment. In addition to this, alternative neurodegenerative disorders and psychiatric disorders must not contribute to language deficits. Lastly, memory and behavioral deficits must not be notable for at least six months, although they may be present further on in the disease course. Upon meeting these criteria, subsequent division into associated PPA variants can be distinguished by neuroimaging and neuropsychological evaluation.

Neuroimaging in navPPA demonstrates atrophy in the left posterior fronto-insular cortex. Positron emission tomography-fludeoxyglucose-18 (PET-FDG) scans within this variant may exhibit hypometabolism in this region of the brain as well. In neuropsychological evaluation, this variant is difficult to assess due to its frequent progression to mutism. Neuroimaging in svPPA demonstrates atrophy of the anterior temporal lobes in the early stages. PET-FDG scans may exhibit hypometabolism in this region of the brain as well. From a neuropsychological standpoint, the assessment of language will demonstrate single-word comprehension deficits and varying levels of comprehension disorders in the form of surface dyslexia and dysgraphia [3].

Motor neuron disease (MND) is a progressive and degenerative process affecting motor neurons that can present with upper motor neuron (UMN) (hyperreflexia, clonus, spasticity, etc.) and/or lower motor neuron (LMN) (atrophy, fasciculations, hyporeflexia, etc.) symptoms. There are various subtypes of MND, with amyotrophic lateral sclerosis (ALS) being the most common form. To be defined as ALS, both UMN and LMN symptoms must be present. ALS can affect the limbs, bulbar muscles, axial skeleton, or respiratory system. Alternative subtypes of MND include primary lateral sclerosis (PLS) and progressive bulbar palsy (PBS). Among the limited body of literature that provides insight into PPA and MND, the subtype of MND that is most often found in this patient population is classical ALS with both UMN and LMN manifestations [4].

The association between FTD and MND has been noted in the literature, although these concurrent presentations are primarily seen among the bvFTD population and very rarely among the PPA population. The presentation of PPA alongside MND was first described in the literature with a patient population of seven cases, although it was not distinguished whether it was PPA or MND that presented first [5]. The clinical value in assessing which of these clinical diagnoses presented initially offers prognostic considerations in this patient population.

In 2019, the prevalence of PPA among patients testing for the FTD-MND diagnosis was assessed in a retrospective study. Of the 32 cases determined to be FTD-MND, 10 cases were determined to be PPA-MND. Of these 10 PPA cases, three were determined to be navPPA, and seven were determined to be svPPA [6]. This study demonstrates that while navPPA is noted in the literature, it represents a rarer clinical population. In other words, there is a clinical population with this diagnosis, and yet there is still little known about disease course and diagnostic/prognostic considerations.

This is the case of a 69-year-old female with navPPA who has a disease course involving MND with functional impairment of daily activities.

## Case presentation

A 69-year-old female presented to the outpatient neurology clinic with her husband, as referred by her psychiatrist. The patient had a history of anxiety and depression, which was managed with sertraline and aripiprazole. In addition, the patient had a past medical history of hyperlipidemia, which was being treated with rosuvastatin. Her husband acted as her primary historian, as she had experienced an insidious and progressive decline in speech over the preceding 18 months. Upon questioning, the patient was noted to respond in one- to two-word phrases, “yes” or “no.” Her husband indicated that prior to these verbal changes, the patient was fully functioning in her job activities and was entirely independent in her activities of daily living. However, upon regression of her language, her husband reported that she was forced to retire. Her husband noted recent changes in short-term memory, although he was unsure if this was due to advanced age or in connection with her language deficits. During a 30-month period, we conducted an assessment of the patient and discovered the following:

Physical exam

Upon initial presentation in the clinic, the patient was noted to have a flat affect, protruded tongue at midline without fasciculations (XII), minimal verbal output, broken speech output, mild left upper extremity bradykinesia, steady gait, reduced arm swing, and atrophy noted on the bilateral hand muscles. Approximately eight months after her initial presentation to the outpatient neurology clinic, the patient was reported to have declined in motor function ability, with a major deficit, including her ability to properly put on her clothing independently. Within 22 months of these initial complaints of motor deficits, the patient was noted to have gait instability without any reported falls.

Neuropsychologic evaluation

The neuropsychologic evaluation was completed approximately 20 months after the initial presentation of the reported speech deficits. Cognitive and emotional aspects of the patient's presentation were assessed, and the conclusion of FTD vs. mixed variant (including Alzheimer's) was made. It was recommended to rule out other etiologies, including delirium vs. rapidly progressive variants of dementia, to create a more complete clinical picture.

Neuroimaging

**Figure 1:**
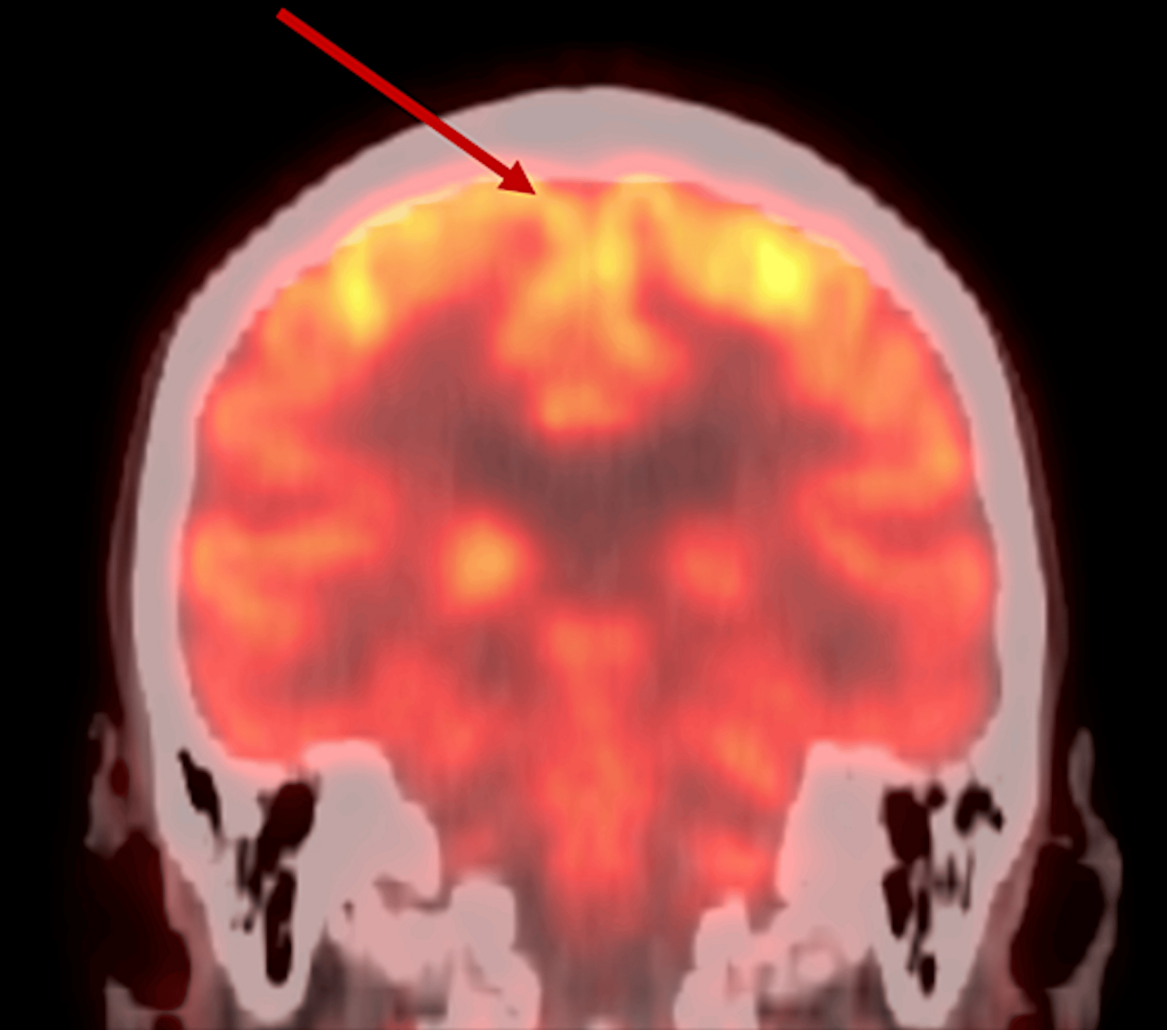
PET scan of the frontal lobe uptake Decreased uptake in the frontal lobes, indicating decreased activity PET: positron emission tomography

MRI scans of the patient's brain completed at 19 and 26 months after the initial presentation of clinical symptoms were stable comparatively and demonstrated "advanced involutional changes with a prominence of the ventricles and sulci as well as severe demyelination in the periventricular deep matter consistent with small vessel ischemic change and/or ischemic leukomalacia." These changes demonstrated bilateral frontal predominant white matter changes. PET-FDG scans of the brain completed 24 months after the initial presentation of the language deficits demonstrated "symmetric decreased FDG uptake in the temporal lobes and to a lesser extent in the frontal lobes bilaterally which may represent frontotemporal dementia." PET-FDG scan is depicted in Figure 1.

Motor studies

Approximately 18 months after the initial functional motor complaints were reported, the patient and her husband agreed to complete motor studies. Prior to the study, atrophy was noted in the bilateral hand muscles. Electromyography (EMG) nerve conduction results revealed abnormal study of the right upper and lower limbs, including normal sensory responses with reduced amplitude of the motor responses. The needle electrode exam showed diffuse changes of denervation and reinnervation in the right upper and lower limb muscles, with an overall concern for MND. Motor nerve conduction studies of the right upper and lower limbs are presented in Table 1.

**Table 1:** Nerve conduction studies/EMG NR: no response; APB: abductor pollicis brevis; ADM: abductor digiti minimi; EDB: extensor digitorum brevis; AH: abductor hallucis; EMG: electromyography

Nerve/Sites	Rec. Site	Lat ms	Amp mV	Rel Amp %	Segments	Dist cm	Velocity m/s
R median-APB							
Wrist	APB	NR	NR	NR	Wrist-APB	7	
Ref.		4.40	4.0		Ref.		
Elbow	APB	NR	NR	NR	Elbow-wrist		
Ref.					Ref.		49.0
R ulnar-ADM							
Wrist	ADM	3.45	7.2	100	Wrist-ADM	7	
Ref.		3.50	6.0		Ref.		
B. Elbow	ADM	7.45	0.9	74.5	B. Elbow-wrist	21	52.5
Ref.					Ref.		49.0
A. Elbow	ADM	9.15	0.9	76.8	A. Elbow-B elbow	10	58.8
R. comm peroneal-EDB							
Ankle	EDB	3.70	1.0	100	Ankle-EDB	9	
Ref.		6.10	2.0		Ref.		
Fib head	EDB	11.25	1.0	96.6	Fib head-ankle	36	47.7
Ref.					Ref.		41.0
Knee	EDB	13.50	0.9	91.1	Knee-fib head	10	44.4
R tibial (knee)-AH							
Ankle	AH	4.85	5.1	100	Ankle-AH	8	
Ref.		6.10	3.0		Ref.		
Knee	AH	13.95	3.0	58.5	Knee-ankle	40	44.0
Ref.					Ref.		41.0

Cerebrospinal fluid analysis

Cerebrospinal fluid (CSF) analysis provided guidance on ruling out possible alternative etiologies. The patient was found to have 0 oligoclonal bands, ruling out concerns for multiple sclerosis. CSF was negative for the venereal disease research laboratory (VDRL) test, ruling out the etiology of neurosyphilis. CSF also resulted negative for the presence of angiotensin-converting enzyme (ACE) and 14-3-3 protein, ruling out neurosarcoidosis and prion disease, respectively. Finally, the Aβ 42/40 ratio was found to be 0.16, indicating a low probability of Alzheimer's.

Counseling

At this point in the clinical timeline, the patient was not of medical decision-making capacity, and her husband was her primary caretaker. After ruling out several possibilities, the patient's husband was informed of the overall concern for PPA, mainly navPPA. Atypically, this was found also to be associated with MND. Resources were provided to the patient's husband, including information on supportive care for the patient and possible ALS support groups. In addition, the patient began follow-up with speech therapy to work on target recall, problem-solving, safety, and expressive language skills.

## Discussion

Meaningful insights into diagnostics and prognostics for patients in the PPA-MND category remain unknown. This case report outlines the clinical association and subsequent clinical testing used to determine an accurate diagnosis among a rare clinical population. The diagnosis of our patient was made primarily by clinical history and physical examination, with supporting evidence from neuropsychological evaluation, neuroimaging, motor studies, and CSF analysis. This patient represents a rare patient cohort in her presentation of navPPA, with clinical signs of MND later in the disease course.

A case of navPPA with progression to MND within two years of diagnosis is noted in the literature, although the initial presentation involving bulbar deficits was significant upon diagnosis. In addition, PET-FDG analysis displayed decreased metabolism in the cingulate gyrus only. Lastly, EMG analysis displayed reduced potentials in bulbar muscles, the trapezius, triceps, and dorsal interossei [7]. The presence of bulbar muscle weakness is different from this current case report, as this factor contributes to the articulation difficulties associated with navPPA rather than the contribution of neurological changes in the PPA diagnosis.

Similar case reports discussing PPA and MND have been noted in the literature, although regarding alternative variants of both PPA and MND. A case of a 49-year-old male diagnosed with svPPA and a co-occurrence of ALS was described in the literature [8]. The symptoms of ALS manifested as weakness, atrophy, fasciculations, hyperreflexia, and spasticity. While this study discussed the semantic variant, our case explores the non-fluent variant. In addition, our patient has exhibited signs of MND, although the variant of ALS was not present.

Prognostic considerations are valuable in counseling patients and their families. The literature showed that patients with concurrent navPPA and MND have survival lengths as low as 24 months versus survival lengths of up to 12 years in those presenting with navPPA only [9]. This supports the finding that the presence of motor neuron dysfunction plays a role in the length of survival [10]. It is valuable to discuss the role that MND has in the progression of disease when counseling patients and their family members in this clinical cohort.

The treatment for PPA is incompletely assessed due to the complexity of progressive degeneration in the disease course. Speech-language therapy has been shown to improve patient outlook in isolated cases, specifically by implementing reading of multisyllabic words and lexical writing therapy. However, these methods of treatment and positive results are not generalizable to the patient population. In addition, transcranial direct current stimulation (tDCS) has been shown to slow the rate of decline in language, although not halting its progression entirely. These positive outcomes are short-lasting and have not demonstrated long-term effects [11]. In a similar manner to PPA, MND is not reversible. Rather, symptoms can be managed. For example, spasticity can be reduced with medications such as tizanidine, gabapentin, or baclofen. In addition, progressive motor symptoms involving dysphagia can be controlled with diet modifications [12]. Counseling measures for family members include discussing plans for advanced care, support groups, and comfort care to improve quality of life. Among the therapies and treatments discussed above, the patient in this case report opted for comfort care, support groups for involved caregivers, and speech therapy.

## Conclusions

NavPPA and its clinical association with MND are rare clinical entities with limited literature on disease progression, diagnosis, prognosis, and counseling measures for caregivers. In this case, a woman in her sixth decade of life presented with language impairments consistent with the navPPA variant of FTD. Within 26 months of the initial language impairments, the patient progressed to a presentation involving motor impairments that interfered with activities of daily living. The diagnostic workup included a thorough clinical history, physical exam, neuropsychological evaluation, neuroimaging, motor studies, and CSF analysis. The line of thorough analysis led to a definitive diagnosis while also narrowing down the differential diagnosis. This clinical presentation offers a guide to healthcare providers on proper medical management for these disease sequelae. Both PPA and MND, as separate clinical entities, are progressive and non-reversible, yet cases such as these offer insight into the limited body of literature that exists. Further discussions on possible treatments to mitigate symptoms of both PPA and MND include speech-language therapy, reading/writing therapy, tDCS, pharmacological management, and lifestyle modifications.
